# Relationships among Mercury Concentration, and Stable Isotope Ratios of Carbon and Nitrogen in the Scalp Hair of Residents from Seven Countries: Effects of Marine Fish and C4 Plants Consumption

**DOI:** 10.1371/journal.pone.0128149

**Published:** 2015-06-12

**Authors:** Tetsuya Endo, Moriaki Hayasaka, Hideki Ogasawra, Osamu Kimura, Yuichi Kotaki, Koichi Haraguchi

**Affiliations:** 1 School of Pharmaceutical Sciences, Health Sciences University of Hokkaido, 1757 Kanazawa, Ishikari-Tobetsu, Hokkaido, 061–0293, Japan; 2 Sapporo Higashi-Tokusyukai Hospital, N33-E14, Higashi-ku, Sapporo, Hokkaido, 065–0033, Japan; 3 School of Marine Biosciences, Kitasato University, Sagamihara, Kanagawa, 252–0373, Japan; 4 Daiichi College of Pharmaceutical Sciences, Minami-Ku, Fukuoka, 815–8511, Japan; New York State Museum, UNITED STATES

## Abstract

We analyzed the Hg concentration, and δ¹³C and δ^15^N values in the scalp hair of residents from seven countries; Vietnam, New Zealand, Spain, the USA, South Korea, Brazil and Japan. Relationships among the data in each country and among the seven countries were then examined. The highest Hg concentration as well as the highest or higher δ^15^N value in each country was found in the hair of a heavy marine fish-eater, whereas the lowest Hg concentration and δ^15^N value were found in the hair of a vegetarian or non (marginal)-fish eater. Hg concentrations were positively correlated with the δ^15^N values in each country, and increased markedly in samples with δ^15^N values exceeding 9.0 ‰, probably due to fish consumption. The highest Hg concentration could be found in sample, with a δ¹³C value between -19 and -18‰, probably reflecting the δ¹³C value of the marine food web.

## Introduction

Mercury (Hg) is distributed throughout the environment via both natural and anthropogenic processes, and Hg toxicity has resulted in widespread public health concerns. The main source of human Hg intake is the consumption of fish and other marine animals contaminated with methyl mercury [1–5]. As Hg accumulation in marine animals increases with increased trophic level, predators accumulate higher levels of Hg [6]-[8].

Hg concentration in scalp hair is the preferred marker for evaluating Hg exposure over a period of several weeks or months, as the hair to blood ratio in humans has been estimated to be about 250:1 [9]. The World Health Organization (WHO) has reported that an Hg level of 50μg/g in human hair corresponds to the “no observed adversary effect level” (NOAEL) for methyl mercury in adults, as determined from neurotoxicological data [9]. In 2003, the Food and Agriculture Organization (FAO)/WHO Joint Expert Committee on Food Additives (JECFA) lowered its guideline value for the provisional tolerable weekly intake (PTWI) of methyl mercury from 3.3 μg/kg-bw/week to 1.6 μg/kg-bw/week [10]. The previous and revised PTWIs correspond to hair Hg levels of 5.0 and 2.2 μg/g, respectively [11]. Most heavy eaters of predatory fish and marine mammals exceed this revised PTWI for methyl mercury [11–13].

Stable isotope analysis has been used as a tool to obtain information on feeding ecology. The stable isotope ratio of nitrogen (δ^15^N) is used to estimate the trophic level of an organism, while the stable isotope ratio of carbon (δ^13^C) is used to estimate the relative contribution to the diet of potential primary sources [14]: a 3 ~ 4 ‰ enrichment in δ^15^N has been shown to occur between the muscle of predators and their prey in wild animals, whereas only a small enrichment is generally found in the δ^13^C value [15], [16]. This enrichment in δ^15^N is the mean value of a significant spread of δ^15^N values between particular consumer-diet pairs. The trophic discrimination values of δ^13^C and δ^15^N were reviewed by McCutchan et al. [17]. Many researchers have reported high levels of δ^15^N in the muscle of marine mammals and predatory fish, reflecting the high trophic positions of the species [7], [18], [19].

Terrestrial plants following the C3 photosynthesis cycle show significantly depleted ^13^C values (about -26 ‰) compared to C4 plants (about -13 ‰) [14]. A large amount of basic human food as well as feed for domestic animals is derived from C3 plants (wheat, barley, soy, potatoes, rice, beans, sugar beets, grass, etc.), although a few species of C4 plants (maize, sugar cane, millet, etc.) are dominant in large regions. In contrast, the δ^13^C values in marine phytoplankton and sea grasses are about -22 ‰ and between -15 and -3 ‰, respectively, and the δ^13^C values in coastal fauna are generally higher than those in pelagic fauna [14]. The shift from coastal or benthic feeding to pelagic feeding, due to the growth of fish, leads to decreased δ^13^C and δ^15^N values [14], [20–22]. To our knowledge, the δ^13^C values in the marine fish reported to date have ranged from -20 to -14 ‰: the δ^13^C values of pelagic fish, such as tuna, swordfish, and sharks, were in the range of -20 to -16 ‰, and those of coastal fish were slightly higher in range, mainly reflecting the trophic positions and the inhabiting areas [7], [19], [23].

Scalp hair and nails are convenient human matrices, as they are stable and easy to collect, transport and store compared to other matrices (e.g., blood and urine). According to previous reports [24–29], δ^13^C in scalp hair samples collected from Europe, Asia, North and South America, and Oceania ranges from -22 to -15 ‰, values which fall between the levels found in C3 plants (about -26 ‰) and C4 plants (about -13 ‰) and similar in range to the levels observed in predatory fish (between -20 and -16 ‰). On the other hand, the level of δ^15^N in scalp hair has been reported to be between 6 and 13 ‰ [24–29], which fall between the levels found in plants (usually between 0 and 5 ‰) [3] and those in marine mammals and predatory fish (generally higher than 12 ‰). As the δ^13^C and δ^15^N values in scalp hair are similar to those in the fingernails (enriched in δ^13^C by ~ 0.2 ‰ and depleted in δ^15^N by ~ 0.6 ‰ compared with fingernail values [30]), the determination of the δ^13^C and δ^15^N values in scalp hair and fingernail samples provides information on the dietary habits and dietary changes from a C3 plant-based diet to a C4 plant-enriched diet as well as from the consumption of animal products to marine products [31].

The consumption of marine products and C4 plant-enriched diets has been found to increase the δ^15^N and δ^13^C values in scalp hair and fingernails, respectively [3], [32]. The enrichment of δ^13^C and δ^15^N values in the scalp hair from diet has been reported to be 2.4 ‰ and 4.5 ‰, respectively [33], which is in contrast with the small δ^13^C enrichment observed in the muscle of wild animals. Macko et al. [33] estimated the dietary habit of ancient humans based on the δ^13^C and δ^15^N values of C3 plant-vegetarians, C4 plant-vegetarians and marine products, as well as the enrichments of δ^13^C and δ^15^N values in scalp hair that result from the diet. Furthermore, the determination of δ^13^C and δ^15^N provides information on geographical location and origin, eating disorders and forensic parameters [3], [30], [32], [34].

The bioaccumulation of methyl mercury in marine animals mainly depends on their trophic level, and positive correlations between δ^15^N values and Hg concentrations in animal muscle have been reported [6–8]. However, the relationships between δ^13^C values and Hg concentrations in wild animals are unclear, probably as δ^13^C is not greatly enriched and the δ^13^C values in plants and marine animals are distributed across a wide range. We previously analyzed the δ^15^N and δ^13^C values as well as the Hg concentration in the scalp hair of whale meat-eaters and non-eaters in Japan, and reported positive correlations between the δ^15^N value and the Hg concentration, and between the δ^13^C and δ^15^N values in the hair of whale meat-eaters [29]. To our knowledge, in spite of many reports concerning δ^15^N and δ^13^C values in scalp hair and many reports concerning Hg concentrations in hair, our previous report was the first to analyze the Hg concentration and δ^15^N and δ^13^C values in the same hair samples. Further study is still, however, necessary to identify the δ^15^N and δ^13^C values and Hg concentration in the scalp hair of heavy fish-eaters.

We collected scalp hair samples from fish-eaters and non-eaters from Vietnam, New Zealand, Spain, South Korea, the USA, Brazil and Japan, and analyzed the Hg concentrations, and δ^13^C and δ^15^N values in the hair samples. We compared the data among the seven countries as well as with the data for whale-meat eaters reported previously [29], and investigated the relationships among the Hg concentration, δ^15^N and δ^13^C values, as well as the frequency of fish consumption.

## Materials and Methods

### Ethic statement

This research project with consenting procedures was approved by the Human Research Ethics Committee of the Graduate School of Pharmaceutical Sciences, Health Sciences University of Hokkaido. Hair donors provided their written informed consent to participate in this study. The principles of Declaration of Helsinki were considered in each part of this study.

### Sampling of scalp hair

Scalp hair samples (about 5 ~ 10 cm in length and 0.5 ~ 1.0 g) from healthy donors were once collected in seven countries, Vietnam, New Zealand, Spain, South Korea, the USA, Brazil and Japan, with the assistance of local collaborators after obtaining written informed consent (Table 1). At the time of hair collection, a simple questionnaire for the collection of data in relation to age, frequency of fish and marine product consumption (per week or day), species of favorite fish, and the daily staple food (rice, wheat, potato, corn, etc.). Eighteen hair samples were collected in Hai Phon and Ha Long, Vietnam, in December 2009. Twenty-one hair samples were collected in Christchurch and Auckland, New Zealand, in February 2010. Twenty-eight and thirty-three samples were collected from Spain (Barcelona) and South Korea (Seoul and its environs) in July-October and September-October 2010, respectively. Twenty-two hair samples were collected in Honolulu, Hawaii (USA), in December 2012, and 31 samples were collected in Salvador, Brazil, in February 2011. The hair donors from each country included heavy fish-eaters who worked as fishmongers, in seafood (Japanese) restaurants or as fishermen. We looked at the fish markets near the hair sampling area in each country to confirm and compare the responses to the questionnaire in relation to the species of fish consumed and consumption levels of fish products.

**Table 1:** Number of hair samples from each of the seven countries and the analytical results.

	No. of samples	Age	Hg (μg/g)	δ^15^N (‰)	δ^13^C (‰)	Frequency
Vietnam	18	39±14	1.6±1.8	9.5±0.7	-20.7±0.7	3.6±2.0
	(m = 11, f = 7)	21–61	0.4–6.9	8.2–10.9	-22.2 to -19.4	0–7
New Zealand	21	27±7.5	2.0±2.5	9.3±0.7	-20.2±0.5	2.2±2.4
	(m = 7, f = 14)	15–45	0.2–10.1	7.6–10.7	-21.3 to- 19.6	0–10
Spain	28	37±14	7.9±12.6	9.7±0.6	-19.8±0.5	3.7±2.2
	(m = 9, f = 19)	20–72	0.1–42.1	8.7–10.8	-20.3 to -18.8	1–8
USA	22	37±20	3.2±5.0	9.3±0.6	-18.3±0.9	7.8±6.8
	(m = 12, f = 10)	6–65	0.2–24.1	7.9–10.5	-20.1 to -16.6	0–21
South Korea	33	42±14	0.8±1.7	9.9±0.9	-18.3±0.8	1.4±2.0
	(m = 17, f = 16)	20–77	0.1–7.2	9.0–12.1	-19.6 to -16.3	0–7
Brazil	31	33±18	2.2±3.1	10.3±0.8	-16.8±0.7	3.2±2.9
	(m = 14, f = 17)	1–70	0.4–15.3	8.7–12.0	-18.3 to -15.8	0–14
Japan	90	37±22	2.6±2.9	9.3±0.5	-18.9±0.6	N.D
	(m = 38, f = 52)	7–88	0.4–18.3	8.6–11.4	-20.2 to -17.6	
Whale meat-eaters^a^	45	60±18	23.415.5	10.2±0.6	-18.4 ±0.5	N.D
	(m = 30, f = 15)	15–87	3.8–67.2	8.9–11.4	-19.5 to -17.2	

The sample numbers of males (m) and females (f) are shown in parentheses.The analytical data are shown as the mean ± SD with range.

^a^Quoted from Endo et al. (23).

Scalp hair samples from 90 donors living in Hokkaido, Aomori, Miyagi and Iwate Prefectures and the Tokyo Metropolitan area, Japan, were collected during November 2009 and September 2013. Most of these Japanese donors consumed fish products, but none consumed products from either toothed whales or dolphins.

All hair samples were packed in polyethylene bags and stored at room temperature until analysis. Prior to analysis, all hair samples were washed twice in a 2:1 chloroform/methanol mixture to remove lipids and other surface contaminants. Washed samples were air-dried and minced by scissors as finely as possible. Table 1 lists the analytical data for the seven countries as well as the data for donors in Japan who ate whale products from toothed whales and dolphins, as reported previously [29].

### Chemical analyses

Total mercury (Hg) concentration in the minced hair samples was determined using a flameless atomic absorption spectrophotometer (Hiranuma Sangyo Co. Ltd., HG-310, Ibaraki, Japan) after digestion by a mixture of HNO_3_, HCO_4_ and H_2_SO_4_ [35]. Human hair No. 13, a certified reference material from NIES (Japan), was used as an analytical quality control for Hg (4.43 ± 0.20 μg/g), and the recovery of Hg was 96 ± 3% (n = 3).

The stable isotope ratios of carbon and nitrogen (δ^13^C and δ^15^N) in the minced hair samples were analyzed using a mass spectrometer (Delta S, Finnigan MAT, Bremen, Germany) coupled with an elemental analyzer (EA1108, Fisons, Roano, Milan, Italy) held in the Center for Ecological Research, Kyoto University, as described previously [36]. δ^13^C and δ^15^N were calculated based on the two-point anchoring method using CERKU-1, -2 and -5, certified by the Center for Ecology Research, Kyoto University [37].

### Statistical analyse*s*

Data were expressed as the mean ± SD. Data were analyzed by Pearson’s coefficient test and multiple linear regression analysis, using the Statcel 2 program, with a value of p<0.05 considered to be significant.

## Results

### Analytical results for hair samples from the seven countries

Table 1 summarizes the age, frequency of fish consumption (per week), and analytical data for Hg concentration, and δ^13^C and δ^15^N values in the scalp hair of residents of the seven countries. The average Hg concentration as well as δ^13^C and δ^15^N values in the male hair from each country were higher, although not significantly, than those in female hair. Table 1 shows the total data for males and females. The data for whale meat-eaters are taken from a previous report [29].

**Fig 1:**
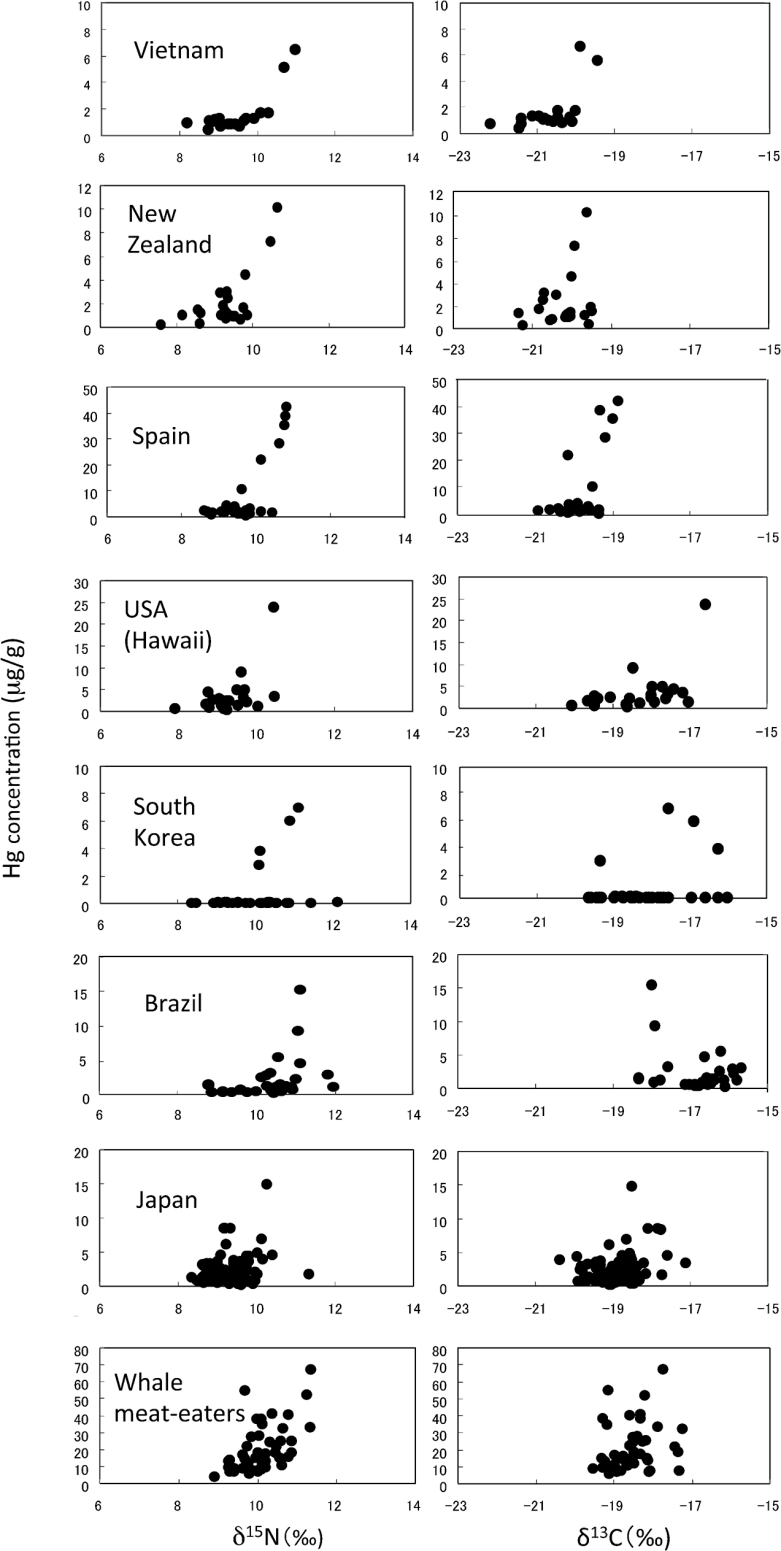
The relationship between Hg concentration and the δ^13^C or δ^15^N value in hair samples from seven countries and Japanese whale meat-eaters.

Fig 1 shows the relationships between Hg concentration and δ^15^N or δ^13^C value in the donors from each country and in the whale meat-eaters. The Hg concentration in the donors from each country as well as in the whale-meat eaters increased markedly with δ^15^N values exceeding 9.0 ‰. The seven countries were classified in three groups according to the average of δ^13^C and the distribution pattern of δ^13^C: the first group included Vietnam, New Zealand and Spain (below -19 ‰), the second group included the USA, Japan and the whale meat-eaters (-21 to -17 ‰), and the third group included South Korea and Brazil (-20 to -15 ‰).

#### Samples from Vietnam

The highest and second highest concentrations of Hg (6.9 and 5.7 μg/g) were found in the hair samples from young brothers who worked as fisherman (21 and 22 years). The δ^13^C and δ^15^N values found in these brothers were -19.9 and 10.9 ‰, and -19.4 and 10.6 ‰, respectively, and these values were the highest and second highest found among the samples from Vietnam. The lowest concentration of Hg (0.4 μg/g) as well as the lowest value of δ^13^C (-22.2 ‰) and a relatively low value of δ^15^N (9.1 ‰) were found in a young woman (25 years) who seldom ate fish products. According to the responses to questionnaires, most of the donors sometimes ate small fish, but not predatory fish such as tuna, swordfish or shark. These predatory fish were not found in the fish markets where the donors lived.

#### Samples from New Zealand

The highest and second highest concentrations of Hg (10.1 and 7.2 μg/g) were found in the hair samples of a manager and cook of Japanese (seafood) restaurants (both male, aged 37 and 45 years), respectively. They also accounted for the highest and second highest values of δ^13^C and δ^15^N (-19.6 and 10.7 ‰, and -19.9 and 10.5 ‰, respectively). Both donors ate predatory fish almost every day. The lowest Hg concentration as well as δ^13^C and δ^15^N values were found in a 43-year-old vegetarian female (0.2 μg/g, -21.3 ‰ and 7.6 ‰, respectively). The average age of the New Zealand donors was the youngest among the seven counties investigated, and the range of ages was the narrowest (Table 1).

#### Samples from Spain

The three highest concentrations of Hg (42.1, 38.6 and 35.2 μg/g) among the samples from Spain were found in the hair from a male fishermen and two male heavy tuna-eaters, all aged in their 50s. The three highest values of δ^13^C and δ^15^N were also found in these donors (-18.8 and 10.8 ‰, -19.3 and 10.8 ‰, and -19.0 and 10.8 ‰). The lowest concentration of Hg (0.1 μg/g) was found in the hair of a female (70 years) who did not eat fish products. This donor also showed a relatively low value of δ^15^N (9.8 ‰) and an intermediate value of δ^13^C (-19.4 ‰).

#### Samples from the USA (Hawaii)

The highest concentration of Hg (24.1 μg/g) among the samples from the USA was found in the sample from a male fishmonger (40 years) who ate marine products at every meal and predatory fish almost every day. This donor also had the highest values of δ^13^C and δ^15^N (-16.6 and 10.5 ‰). More than half of the donors ate predatory fish at least once a month and showed Hg concentrations above 2.0 μg/g. The lowest concentration of Hg (0.2 μg/g) was found in the hair of a male donor (47 years) who scarcely ate fish. This donor also showed intermediate values of δ^13^C and δ^15^N (-18.61 ‰ and 9.26 ‰, respectively) in comparison with the other donors. The lowest δ^13^C and δ^15^N values (-20.1 and 7.9 ‰) were found in the hair from a female donor (56 years) who did not eat fish products, and the Hg concentration in this donor was 0.4 μg/g.

#### Samples from South Korea

The highest and second highest concentration of Hg (7.2 and 6.3 μg/g) among the South Korean samples were found in the hair of two male managers of Japanese (seafood) restaurants (60 and 57 years) who ate fish products almost everyday. These donors also showed relatively high values of δ^15^N (11.1 and 10.9 ‰) and δ^13^C (-17.6 and -16.9 ‰). The average Hg and frequency of fish consumption were the lowest among the seven countries investigated. The Hg concentrations in most of the South Korean samples were below 0.5 μg/g. The δ^15^N and δ^13^C values in some donors exceeded 10 ‰ and -17 ‰, but the Hg concentrations in these donors were trace, which is in contrast with the Hg distribution pattern of the other countries (Fig 1). According to the responses to the questionnaires, most of the donors sometimes ate small fish, but not predatory fish such as tuna, swordfish or shark. We seldom found predatory fish in the markets where the donors shopped.

#### Samples from Brazil

The highest concentration of Hg (15.3 μg/g) was found in the hair of a female fishmonger (60 years) who frequently ate fish products. This donor also showed a relatively high value of δ^15^N (11.1 ‰) and a relatively low value of δ^13^C (-18.0 ‰) compared to the other samples from Brazil. Some of the donors occasionally ate predatory fish, particularly shark, and high concentrations of Hg were found in their hair. The lowest concentration of Hg (0.4 μg/g) was found in the hair sample from a female donor (20 years) who seldom ate fish products. This donor also showed the lowest value of δ^15^N (8.7 ‰) and an intermediate value of δ^13^C (-16.9 ‰). The average δ^13^C (-16.8 ‰) and δ^15^N (10.3 ‰) values from the Brazilian samples were the highest among the seven counties investigated, although the average Hg concentration was at an intermediate value (Table 1).

We analyzed the δ^13^C and δ^15^N values and Hg concentration in shark muscle randomly purchased from supermarkets. The δ^13^C values from five shark samples were in the range of -17.5 to -15.2 ‰, the δ^15^N values were in the range of 9.8 to 13.4 ‰, and the Hg concentrations were in the range of 0.4 to 3.02 μg/g. The δ^13^C values found in the shark muscle were negatively correlated with the Hg concentrations (γ = 0.941, p<0.05).

#### Samples from Japan

The highest concentration of Hg (18.3 μg/g) among the Japanese samples was found in the hair from a male fishmonger (80 years) who frequently ate predatory fish. This donor also showed relatively high values of δ^13^C (-18.1 ‰) and δ^15^N (10.4 ‰). The lowest concentration of Hg (0.4 μg/g) was found in the hair from a female donor, aged 35 years, who seldom ate fish products. She also showed intermediate values of δ^13^C (-19.0 ‰) and δ^15^N (9.6 ‰) compared with the other Japanese samples.

According to our previous report [29], the average value of δ^15^N in whale meat-eaters was significantly higher than those in non-eaters (shown in Table 1). The highest Hg concentration among the whale meat-eaters was 67.2 μg/g, with the second highest value of δ^15^N being 11.3 ‰. The δ^15^N and δ^13^C values of the cetaceans and predatory fish consumed were between 9 and13 ‰ and between -16 and -18 ‰, respectively. The average concentration of Hg in the hair of whale meat-eaters (23.4 ± 15.5 μg/g, n = 45) was about nine times higher than that of non-eaters (2.6 ± 2.9 μg/g, n = 90). The average age of whale meat-eaters (60 ± 18 yr, n = 45) was significantly older than that of non-eaters (37 ± 22 yr, n = 90) and of the hair sample donors from other countries.

### Correlations among δ^13^C and δ^15^N values and Hg concentration, and between Hg concentration and age or frequency of fish consumption in the samples from each country

Table 2 lists the correlation coefficients (γ) among Hg concentration, δ^15^N and δ^13^C values, and between Hg concentration and age or frequency of fish consumption per week.

**Table 2:** Correlation coefficients among Hg concentration, andδ^13^C andδ^15^N values, and between age and Hg concentration shown in Table 1.

	Number of samples	Hg vs δ^15^N	Hg vs δ^13^C	δ^13^C vs δ^15^N	Age vs Hg	Hg vs frequency
Vietnam	18	0.748*	0.607*	0.409	0.410^a^	0.592*
New Zealand	21	0.677*	0.261	0.311	0.396	0.636*
Spain	28	0.763*	0.584*	0.692*	0.432*	0.746*
USA	22	0.518*	0.493*	0.620*	0.122^b^	0.437*
South Korea	33	0.321	0.313	0.638*	0.699*	0.785*
Brazil	31	0.362*	- 0.448*	0.284	0.326	0.279
Japan	90	0.372*	0.248*	0.305*	0.406*	N.D
Whale meat-eaters^c^	45	0.549*	0.202	0.422*	0.227	N.D

*Significant correlation (p<0.05)

^a^Two samples with Hg concentrations of 6.9 and 5.7 μg/g were deleted (n = 16).

^b^One sample with an Hg concentration of 24.1 μg/g was deleted (n = 21).

^c^Quoted from Endo et al. (23)

The δ^13^C values in the hair samples from Spain, South Korea, the USA and Japan were positively correlated with their δ^15^N values (p<0.05), but no significant positive correlation was found in samples from Vietnam, New Zealand and Brazil.

The Hg concentrations in the hair samples from Vietnam, New Zealand, Spain, the USA, Brazil and Japan were positively correlated with their δ^15^N values (p<0.05), but this correlation was not significant in samples from South Korea (p<0.10). Similar or slightly higher correlations (γ) were found between the logarithmic concentrations of Hg and the δ^15^N values.

It is worthy of note that the δ^13^C value in the Brazilian samples was negatively correlated with the Hg concentration (p<0.05), which was in contrast with the tendency towards positive correlations found in the other countries (Table 2).

Significant positive correlations (p<0.05) were found between Hg concentration and the age of donors from Vietnam, Spain, South Korea and Japan, and positive tendencies were observed in the donors from New Zealand, the USA and Brazil (p>0.05). However, no correlation was found between the age and δ^13^C value or δ^15^N value in the combined donors from the seven countries (data not shown in Table 2).

Significant positive correlations (p<0.05) were found between the Hg concentration and frequency of fish consumption (per week) of donors from all countries except for Brazil (Table 2). Although the frequency data was not available for all Japanese donors, the Hg concentrations in nineteen donors increased significantly with increased frequency of consumption (p<0.05, data not shown in Table 2).

The Hg concentrations in the donors from the seven countries and whale meat-eaters were analyzed by multiple linear regression analysis (Table 3). This analysis showed that the Hg concentrations of the donors from all countries, except for the USA and Brazil, had significant positive correlations with at least one of parameters examined; δ^15^N value, δ^13^C value, age and frequency of fish consumption. In contrast, the Hg concentration in the donors from the USA tended to show a positive relationship with the δ^13^C value, whereas that from Brazil tended to show a negative relationship with the δ^13^C value.

**Table 3:** Standardized partial coefficients for the seven countries and whale meat-eaters from the multiple linear regression analysis of Hg concentration.

	Vietnam	New Zealand	Spain	USA	South Korea	Brazil	Japan	Whale meat-eaters
δ^15^N	0.498*	0.385*	0.406*	0.286	0.027	0.232	0.243*	0.527*
δ^13^C	0.298	0.202	0.120	0.355	0.039	-0.343	0.169	-0.005
Age	0.012	0.453*	0.049	0.168	0.227	0.268	0.350*	0.128
Frequency	0.205	0.446*	0.455*	0.002	0.773*	0.138	—-	——

* p<0.05

### Correlations among δ^13^C and δ^15^N values and Hg concentration in the combined samples from the seven countries

**Fig 2:**
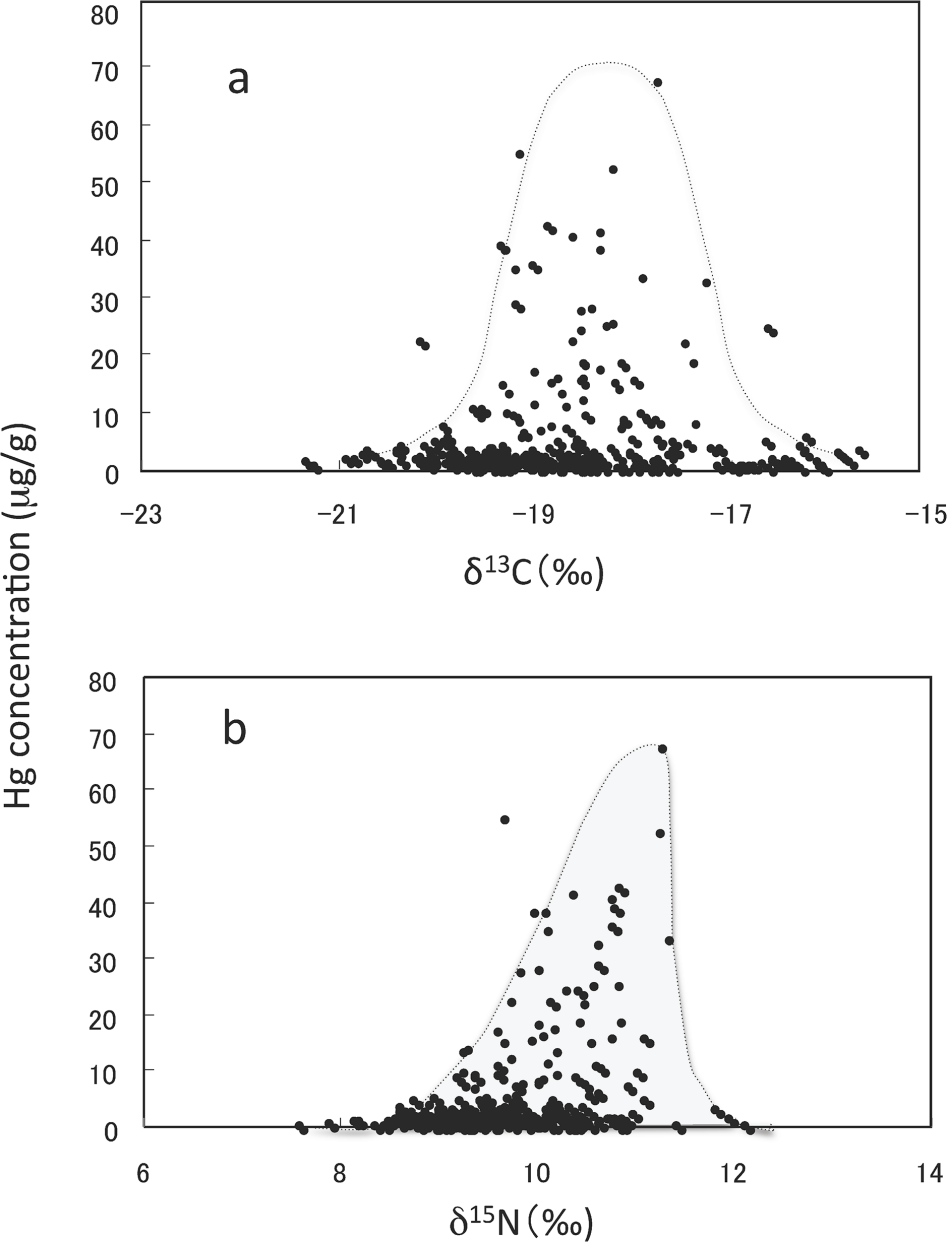
The relationship between Hg concentration and the δ^13^C or δ^15^N value in the combined hair samples from seven countries and Japanese whale meat-eaters.

Fig 2A shows the relationship between the δ^13^C value and Hg concentration in the combined samples from the seven countries and the whale meat-eaters (n = 288). The peak of the curve for δ^13^C appears to be between -19 and -18 ‰.

Fig 2B similarly shows the relationship between the δ^15^N value and Hg concentration in the combined samples from the seven countries and the whale-meat eaters (n = 288). The Hg concentration increased with δ^15^N values up to about 11.5 ‰, but further increases in Hg could not be confirmed as only five samples showed δ^15^N values exceeding 11.5 ‰ (the highest value was 12.1 ‰).

**Fig 3:**
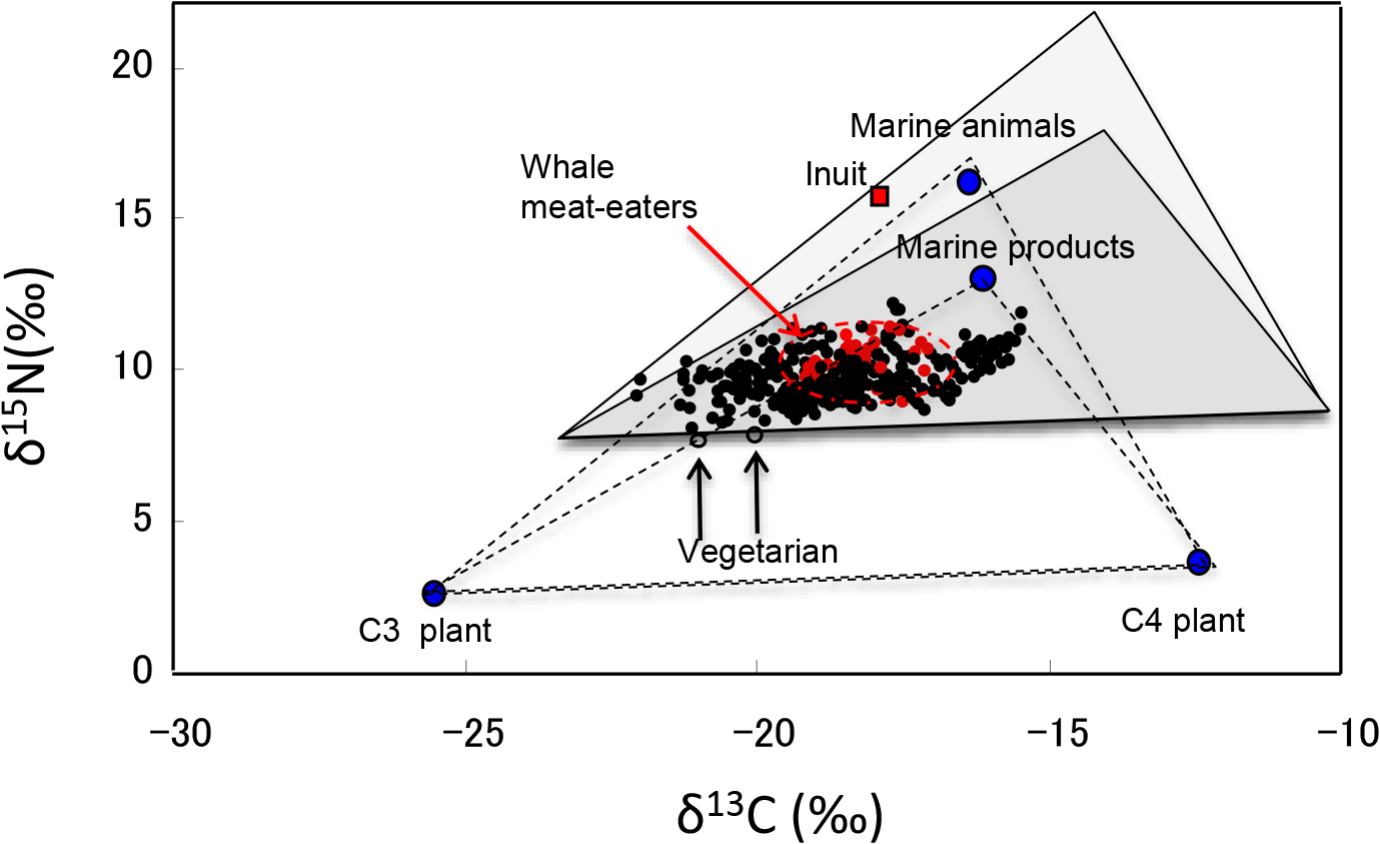
Isotope analyses of carbon and nitrogen in the combined hair samples from donors from seven countries, Japanese whale meat-eaters and the Inuit.

Fig 3 shows the relationship between the δ^13^C and δ^15^N values in the combined samples from the seven countries and the whale meat-eaters (n = 288). Triangles in the figure link the average values for C3 plants, C4 plants and marine products (unbroken lines) or marine animals (dashed lines). The average δ^13^C and δ^15^N values in the C3 plants, C4 plants and marine products used were -25.7 and 2.5 ‰, -12.3 and 3.5 ‰, and -16.3 and 12.5 ‰, respectively [31], and those of marine animals were -16.5 and 16.0 ‰, respectively [3]. To estimate dietary habits, these triangles were shifted toward the top right corner to correct for δ^13^C and δ^15^N enrichments [34]. The enrichment values of δ^13^C and δ^15^N from the diet to the hair used were +2.4 ‰ and +4.5 ‰, respectively [33]. The shifted triangles are shown in gray.

Most of the hair samples from the seven countries and from the whale meat-eaters were distributed over the central region from the left edge of gray triangle shown by the unbroken lines. The δ^13^C and δ^15^N values of the two vegetarians from New Zealand and the USA were located almost on the unbroken line connecting the C3 plants and C4 plants, and most of the samples from Brazil and South Korea were distributed across the central region.

Furthermore, we also estimated the dietary habits of Inuit using the δ^13^C and δ^15^N values of marine animals eaten this ethnic group. The average values of δ^13^C and δ^15^N from the hair of Inuit (-18.0 and 15.4 ‰, respectively), estimated from the fingernail data provided by Buchardt et al. [3], were distributed along the dashed line connecting the C3 plants and marine animals, which was just above those of the whale meat-eaters.

## Discussion

To our knowledge, the δ^13^C and δ^15^N values in scalp hair reported in most countries are distributed between -22 ‰ and -15 ‰ and between 6 ‰ and 13 ‰, respectively [24–28]. The δ^13^C and δ^15^N values in the combined samples from the seven countries (n = 243) and in the whale meat-eaters (n = 45) shown in Table 1 were distributed widely across the reported ranges of δ^13^C and δ^15^N. The Hg concentrations in the hair samples from donors from the seven countries and from the whale meat-eaters ranged widely between 0.1 to 67.2 μg/g (n = 288). Among the donors from the seven countries (n = 243), the Hg concentration exceeded 2.2 μg/g (PTWI of methyl mercury) in 58 donors, and exceeded 5 μg/g (the previous PTWI) in 26 donors, but no donor showed an Hg concentration exceeding 50 μg/g (NOAEL). Many studies have reported similar Hg concentrations in the hair of fish-eaters [2], [3], [11], [13], [38].

The donor with the highest Hg concentration and the highest or relatively high value of δ^15^N in each country was a heavy fish-eater. The Hg concentrations in the hair of non-fish eaters in the present study were about 0.4 μg/g or lower (Table 1), and increased with the frequency of fish consumption (Table 2). In agreement, Petzke et al. [31] reported a positive correlation between δ^15^N value in hair and fish consumption, and Elhamri et al. [2] reported that the Hg concentration in the hair of non-fish eaters and marginal fish-eaters seldom exceeded 0.4 μg/g. As shown in Table 2, the Hg concentration in the hair increased with increased δ^15^N value. One notable result was that the Hg concentrations in the hair of donors, probably of fish-eaters, increased markedly when δ^15^N values exceeded 9.0 ‰, but further increases in Hg concentration when the δ^15^N value exceeded 11.5 ‰ could not be confirmed due to the limited data available for the higher δ^15^N values (Fig 2B). On the other hand, the Hg concentration in the hair could be the highest when the δ^13^C values ranged between -19 and -18 ‰ (Fig 2A). The δ^13^C values reported in marine predators ranged from -20 ‰ to -16 ‰ [6], [18], [29], while those in marine animals captured in the Brazilian coastal waters were slightly higher than this range [7]. The peak δ^13^C value shown in Fig 2A may reflect the δ^13^C values of predatory fish as well as the intermediate values of C3 plants and C4 plants (-26 ‰ and -13 ‰). The present report is the first study to clarify the relationship among Hg concentration, δ^15^N and δ^13^C values in scalp hair. One exception to the above conclusion was that the δ^15^N values in five donors from Brazil (two adults), South Korea (two adults) and Japan (an infant) exceeded 11.0 ‰, although the Hg concentrations in those donors were trace. The high δ^15^N values in breast-fed infants [24], [39] may explain the high δ^15^N value found in the Japanese infant. Possible reasons for the high δ^15^N values found in the adults are that grey hair (lacking pigment) contains undetectable amounts of Hg [40], artificial waving decreases the Hg concentration [41], or that dyeing and bleaching of scalp hair affects the analysis of δ^15^N and δ^13^C values whereas the lack of pigment does not [39]. Furthermore, the potential influence of urban pollution of δ^15^N [42] cannot be ruled out for the cause of high δ^15^N values in the donors.

The present survey included two vegetarians. The δ^13^C and δ^15^N values of the samples from vegetarian from New Zealand were -21.3 ‰ and 7.6 ‰, respectively, and from the vegetarian from the USA were -20.1 ‰ and 7.9 ‰, respectively. The Hg concentrations in those vegetarians (0.2 and 0.4 μg/g) were the lowest among the New Zealand samples and the second lowest among the USA samples. In agreement with the present data, most of the previously reported δ^15^N values in vegans were distributed between 6 and 8 ‰ [24], [34], [39], the Hg concentrations in the scalp hair of vegetarians were markedly lower those in omnivores [43], and the Hg concentrations in the hair of non-fish eaters seldom exceeded 0.4 μg/g [2].

The average values of δ^13^C and δ^15^N in the samples from Brazil were the highest among the seven countries. The four highest δ^13^C values in the Brazil samples were -15.7, -15.8, -15.8 and -15.9 ‰, but the Hg concentrations in those four samples (1.2 to 2.9 μg/g) were close to the average value for the Brazil samples. It is worthy of note that the δ^13^C values in the Brazil samples were negatively correlated with their Hg concentrations (p<0.05), while the δ^13^C values in the other countries were positively correlated (Table 2). According to the multiple linear regression analysis (Table 3), the Hg concentration more closely associated with the δ^13^C value, although negatively, than with the δ^15^N value, age or frequency of fish consumption (positively). The scatter plot of δ^13^C versus Hg concentration for Brazilians (Fig 1) may be typical of donors consuming a C4-plant-enriched diet. High levels of δ^13^C were reported in the scalp hair and fingernails of Brazilians [25], [26], although the Hg concentrations in those tissues were not determined. Interestingly, the δ^13^C values found in the shark muscle sold in the Brazilian markets were negatively correlated with the Hg concentrations (p<0.05). Bisi et al. [7] investigated the relationships between δ^15^N value and Hg concentrations and between δ^13^C and δ^15^N values in marine animals inhabiting the Brazilian coastal waters, and reported that the δ^15^N value was positively correlated with the logarithmic concentration of Hg. However, they did not analyze the relationship between the δ^13^C value and Hg concentration.

Fig 3 shows triangles generated from the average δ^13^C and δ^15^N values of C3 plants, C4 plants and marine products. The samples from Brazil and South Korea were distributed near the center of the shifted triangle (gray), and other samples were mainly distributed in the corner of the shifted triangle. These results suggest that the donors from Brazil and South Korea consume C4 plant-enriched diets whereas the donors from the other countries mainly consume C3 plant-based diets. Interestingly, the δ^13^C and δ^15^N values of the two vegetarians were located almost on the solid line connecting the C3 plants and C4 plants, which is in agreement with their dietary habits.

We estimated the dietary habits of Inuit in the same manner as described above using the δ^13^C and δ^15^N values of marine animals eaten by this ethnic group. The estimated values of δ^13^C and δ^15^N in the Inuit hair were distributed along the dashed line of the gray-shaded triangle (connecting C3 plants and marine animals). This result is in good agreement with the fact that the Inuit diet consists of marine animals and C3 plants in a 1:1 ratio. To estimate dietary habits, we used the values of δ^13^C and δ^15^N in the C3 and C4 plants and marine products reported by Huelsemann et al. [31], and the enrichments in δ^13^C and δ^15^N values in scalp hair reported by Tokui et al. [33]. Similar δ^13^C and δ^15^N values in foods were reported by Nardoto et al. [25] and similar values for the enrichments from the diet to the hair were reported by Yoshinaga et al. [44].

The estimated δ^15^N value in the hair of the Inuit (15.4 ‰) was markedly higher than the reported values of δ^15^N in the heavy fish-eaters and whale meat-eaters reported by many investigators (lower than 13 ‰), whereas the estimated δ^13^C value in the Inuit corresponded to the average value of the whale meat-eaters (Table 1 and Fig 3). The Hg concentrations in the hair of Inuit could not be estimated from Fig 2A because no hair samples in this survey showed a δ^15^N value exceeding 12.1 ‰. According to Weihe et al. [12], the average Hg concentration in the maternal hair of the Inuit is 15.5 μg/g (n = 31) and that in the hair of children is 5.5 μg/g (n = 43). The δ^15^N values in the marine mammals consumed by the Inuit may be higher than those by the whale-meat eaters (9 ∼ 13 ‰) [29]. Analyses of Hg concentration, δ^13^C and δ^15^N values in the same hair samples from Inuit donors in addition to their food are necessary to clarify the relationships among these parameters.

As we did not select donors at random from the general population and the sample numbers were limited, the present data may not be representative of the population of each country, except for Japan (n = 90). The average Hg concentration in the scalp hair from Spain previously reported was about 1.0 μg/g [5], which is in contrast to the present data (7.9 ± 12.6 μg/g). On the other hand, the Hg concentrations in scalp hair samples from South Korea previously reported [38], [45], [46] were similar to the present data. Unfortunately, previous studies on δ^13^C and δ^15^N in scalp hair from Spain and South Korea are lacking. The average δ^13^C and δ^15^N values in the hair from the central USA were reported to be -16.8 ± 0.8 ‰ and 8.8 ± 0.4 ‰ (n = 206), respectively [28], and the average Hg concentration was reported to be below 1.0 μg/g [1]. The present data for δ^13^C values in the USA (Hawaii) were slightly lower, whereas the present data forδ^15^N values and Hg concentrations were higher than those previously reported for the USA. The δ^13^C and δ^15^N values in the Brazilian samples were the highest among the seven countries (Table 1). In agreement with our data, a high level of δ^13^C was reported in the scalp hair [26] and fingernails [25] from Brazilian donors. Not only their C4 plant-enriched diet but also geographical factors may contribute to these high δ^13^C and δ^15^N values. Many research groups have reported high concentrations of Hg in scalp hair due to the consumption of Hg-contaminated freshwater fish caught off the gold mining areas in the Amazon, Brazil [47]. However, little is known about the relationship among the Hg concentration, and δ^13^C and δ^15^N values in hair samples from donors who ate freshwater fish contaminated with Hg from anthropogenic origins. Further, no information is available on the Hg concentration, and δ^13^C and δ^15^N values in the scalp hair from Vietnam and New Zealand. The present data for Hg concentrations in hair samples from Japan (n = 90) are in good agreement with those of large scale surveys in Japan (n = 8665) reported by Yasutake et al. [11]. Unfortunately, there is an absence of large scale surveys of δ^13^C and δ^15^N in hair samples taken from Japanese. The Hg concentration in the hair of Japanese donors (n = 90) was found to increase with age (Table 2), and a similar tendency was observed in the donors from the other countries. We speculate that the Hg concentration in the hair of fish-eaters tends to increase with age.

In conclusion, we analyzed hair samples from donors from seven countries. The highest Hg concentration in each county was found in the hair of heavy fish-eaters. These donors also showed the highest or relatively high values of δ^15^N. The lowest Hg concentrations and the lowest δ^15^N values were found in the hair of vegetarians or non (marginal)-fish eaters. The average δ^13^C and δ^15^N values in hair from Brazilian donors were the highest, probably reflecting not only their dietary diversity but also their geographic diversity. The highest Hg concentration in hair samples was found in the samples with a δ^13^C value ranging from between -19 and -18 ‰, probably reflecting the δ^13^C value of the predatory fish consumed. The Hg concentrations in hair samples tended to increase markedly with δ^15^N values exceeding 9.0 ‰, which could reflect high levels of fish consumption. Not only dietary and geographical diversities, but also the nutritional and metabolic status of the donor affect the δ^13^C and δ^15^N values in hair [32], and increased age and frequency of fish consumption increase the Hg concentration. Further well-designed studies are necessary to confirm and develop the present findings.
